# Exogenous Endophthalmitis in Diabetic Patients: A Systemic Review

**DOI:** 10.5402/2012/456209

**Published:** 2012-10-17

**Authors:** Georges M. El-Mollayess, Joanna S. Saadeh, Haytham I. Salti

**Affiliations:** The Department of Ophthalmology, American University of Beirut Medical Center, P.O. Box 11-0236, Beirut 1107 2020, Lebanon

## Abstract

Diabetes mellitus is a systemic disease that increases the risk of infections. Exogenous endophthalmitis is an inflammatory disease to which diabetic patients are more predisposed to than nondiabetic patients undergoing any intraocular intervention. This might be because of the change in the immune and inflammatory factors that intervene in wound healing and in the bacterial flora of the ocular adnexa. We conducted a literature review to assess the risk of exogenous endophthalmitis in diabetic patients undergoing cataract extraction, pars plana vitrectomy, and intravitreal injections and to check whether its treatment differ from in non-diabetics. We found that diabetic patients are more predisposed to virulent organisms and that the incidence of ophthalmic symptoms was not substantially different in diabetic versus nondiabetic patients. Regarding treatment, all patients with light perception should receive pars plana vitrectomy, while those with hand motion and better vision should be given an intravitreal antibiotics injection. Some authors recommend vitrectomy to diabetic patients with even counting figure vision.

## 1. Introduction

Infectious endophthalmitis is defined as the infestation of the intraocular compartment by microorganisms. It represents one of the most severe and potentially devastating inflammatory reactions seen in the eye and it often results in irreversible visual loss [1, 2]. Based on the site of entry of this agent, endophthalmitis can be divided into an infection of either exogenous or endogenous origin. While the former condition most commonly occurs after intraocular surgery or trauma, the latter form is believed to be linked to septicemia. Postoperative bacterial endophthalmitis is a rare, but severe complication of intraocular surgery. The infecting bacteria by replication and release of toxins can damage the intraocular structures, and the inflammatory reaction of the host seems to contribute even further to the damage [3].

Patients with diabetes mellitus (DM) known to have an impaired immune response may be at a higher risk for developing postoperative infections. Effectively at the cellular and humoral levels, there is suboptimal response to different antigens in addition to altered phagocytic capabilities [4–7]. Moreover, it is established that impaired neutrophil bactericidal function is strongly associated with poor glycemic control [8, 9]. Furthermore, the tear film, the first immunological barrier for the ocular system, is altered in patients with DM. There is documented decrease in the breakup time and an established decrease in Schirmer's test [10]. Finally, it has been demonstrated that diabetes mellitus alters the corneal epithelial basement membrane resulting in basal cell degeneration manifested clinically as a superficial punctate keratitis and breakdown of the barrier function of this front line epithelium resulting in greater fragility of the eyeball [11–17].

These deficiencies in the protective features inherent to the eye seem to all associate with the duration of the disease and the serum level of glycosylated hemoglobin HbA1c [18, 19]. Elevated levels of of glucose seem to impair epidermal growth factor receptor (EGFR) signaling and suppress basal cell multiplication and wound-induced AKT phosphorylation [20–22]. This anomaly results in delayed healing of the epithelial defect, which may be associated with sight-threatening complications, such as stromal opacities, surface irregularities, microbial keratitis, and increased risk of acute postoperative endophthalmitis due to delayed and improper wound closure [22]. Independent of all the above and with the raised levels of glucose in the skin, mucous membranes and the tear film of patients with DM, microorganisms growth seems definitely promoted.

## 2. Epidemiology

The reported incidence of postoperative endophthalmitis varies by the specific surgical procedure, but overall the occurrence has declined substantially in the past century. The incidence of endophthalmitis after cataract surgery has decreased from approximately 10% in the late 1800s to 0.58% in the mid-1900s to 0.09% in the early 1990s to 0.04% during the period 1995 to 2001 [1, 23–26]. Rates of endophthalmitis after conventional pars plana vitrectomy (PPV) have further decreased over the past 25 years. Since Ho and Tolentino reported an endophthalmitis rate as high as 0.15% after PPV in 1984, in subsequent years, the incidence has decreased to a range from 0.018% to 0.07% [25–32]. The question that whether sutureless vitrectomy increases the risk of endophthalmitis is still controversial [31, 33, 34]. Past literature suggests that subjects with diabetes have an increased tendency, after cataract surgery, to develop endophthalmitis [1, 32, 35, 36]. A plethora of reports show that approximately 14 to 21% of postoperative patients who develop endophthalmitis are diabetic [37–40]. 

## 3. Symptoms and Presentation

The incidence of ophthalmic symptoms was not substantially different in diabetic versus nondiabetic subjects, especially that patients presenting with ocular pain are nearly as frequent in both types of subjects: the diabetic and the nondiabetic. The median number of days from onset of symptoms to presentation also did not differ in both types of patients (on the average 4 days) [38]. The differences were essentially a trend towards a more opaque media in diabetic subjects upon examination than in the nondiabetics. A retinal vessel could not be seen with indirect ophthalmoscopy at presentation in 90% of diabetics, versus 77% in nondiabetic patients. Also, the incidence of rubeosis at presentation was 8.6% in diabetics versus 1.9% in the nondiabetics [38].  Table 1 summarizes the frequency of symptoms in diabetic patients and in nondiabetic patients.

## 4. Types of Isolated Microorganisms and Antibiotics Sensitivity 

Postoperative endophthalmitis is most often associated with gram-positive organisms (75%–80%), next in frequency are gram-negative organisms (15%–29%), and least often fungi (3%–13%). When cultures were obtained from nondiabetic patients they were likely to show no growth in as many as twice the number noted in cultures from diabetic subjects that is at 33% versus 15%, respectively. In addition, the microorganisms that grew from eyes of diabetic subjects had a preponderance of more virulent organisms at 26% versus 22% isolated from eyes in nondiabetic patients [38]. The most common organism isolated from diabetic patients with acute endophthalmitis is coagulase-negative *Staphylococcus* [37]. In another study, both type 1 and type 2 diabetic patients grew *staphylococcus epidermis* and *staphylococcus aureus* at 11.79% and 11.7%, respectively for type 1, and 24.2% and 21.2% for type 2 [10]. There was a trend for a higher preponderance of coagulase-negative staphylococcal isolates in patients who had preexisting diabetic retinopathy [10]. Phillips and Tasman found a higher prevalence of gram-negative isolates of *Staph* organisms among their patients. Remarkably none of their patients had gram-negative endophthalmitis [41]. Of note, is the conjunctival flora of diabetic subjects differed from that isolated from nondiabetic subjects [10]. 

As for resistance to antibiotics, very few reports exist in the literature comparing resistance to antibiotics among different microorganisms isolated from patients with diabetes as opposed to those without. On the one hand, resistance was observed and reported to penicillin, ampicillin, and tetracycline in *S. aureus* isolates, on the other hand resistance to vancomycin was absent, thus singling out this antibiotic as the most effective therapeutic option [10].

## 5. Treatment

**Figure 1:**
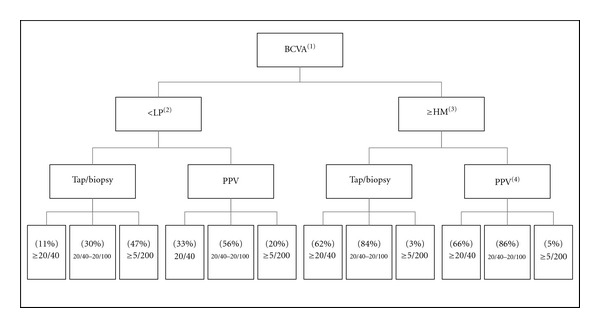
Visual outcome of endophthalmitis vitrectomy study. (1) Best corrected visual acuity, (2) light perception, (3) hand motion, (4) pars plana vitrectomy.

Endophthalmitis still poses a challenge to both the timing and the modality of treatment. To date, the largest series that has directed the standard of care remains to be the EVS, which in summary recommends the following. In eyes with LP vision or better, tap and injection were as favored as the use of pars plana vitrectomy plus tap. While in eyes with LP vision or worse the group that underwent the PPV fared better than patients who received tap with intravitreal antibiotics only. Subanalysis of the patients with diabetes in the EVS cohort was inconclusive although it suggested a more favorable outcome for patients who underwent a vitrectomy regardless of their visual outcome [42] (Figure 1: summary of the study is included in the flowchart).

The use of intravitreal dexamethasone along with the antibiotics injection is still controversial. On the one hand, if intravitreal steroids are given early enough in the course of endophthalmitis, there might be some benefit for better outcome in endophthalmitis through a reduction in the inflammatory response to the infectious organism [43–46]. In one study done on nondiabetic patients who developed endophthalmitis, there was a trend towards a better visual outcome in patients with suspected bacterial endophthalmitis when treated with a combination of intravitreal antibiotics and dexamethasone [47]. On the other hand, another report demonstrated that intravitreal steroids may not be effective for acute endophthalmitis besides and it is important to be cautious using it, keeping in mind its potential toxicity and blunting of the immune response that is necessary to combat infections [48]. Jett et al. noted that combining intravitreal steroids with antibiotics may have beneficial effects in eyes infected with only bacteria that are nontoxin-producing and are less virulent; they reported that manipulation of the timing of dexamethasone administration did not have any significant effect on the treatment outcome [49]. It is obvious that studies on the use and timing of intravitreal steroids in diabetic patients with endophthalmitis are badly needed to establish a solid basis for treatment that would constitute standardized care.

## 6. Systemic Antibiotics

Prior results from EVS demonstrated that systemic antibiotics were not effective in the management of acute postcataract extraction endophthalmitis [40]. 

## 7. Visual Outcome after Treatment

The outcome of vision in patients with diabetes treated for endophthalmitis is usually less successful than in patients with no diabetes. Phillips and Tasman found that 26% of patients with diabetes achieved a final postoperative visual acuity equal or slightly more than 20/200 in contrast to 59% in patients without diabetes. Additionally, 30% of these diabetic patients failed to have not even light perception vision [41]. In an exploratory analysis among patients in the Endophthalmitis Vitrectomy Study (EVS), 58 out of 420 (13.8%) eyes of diabetic patients were analysed over a 9-month period, 80% achieved visual acuity of 5/200 or greater in contrast to 90% in patients who had no diabetes. Along this trend, 55% and 39% of subjects with diabetes achieved a final visual acuity equal or slightly greater than, respectively, 20/100 or greater and 20/40 or greater, compared to 77% and 55% in nondiabetic patients, respectively. As to patients who presented with better than LP vision, diabetic subjects achieved 20/40 more often when vitrectomy was applied (57%) than with tap/biopsy (40%) [38, 40]. No operative complications were observed in either group although early postoperative interventions were significantly higher in the group of diabetic patients (20.7%). However, the statistical power is very low because of the small number of subjects with diabetes [38]. Thus, until a large clinical trial studying treatment outcome in diabetic patients with better than LP vision is done, it is recommended that initial vitrectomy or tap/biopsy are reasonable approaches for diabetic patients with better than LP vision [50]. 

## 8. Endophthalmitis and Intravitreal Injection

Endophthalmitis, although a rare complication of intravitreal injection, is a serious and clinically relevant concern. This is because there are situations in which the frequency and number of injections are obligatory to avoid potential loss of vision, which otherwise may be permanent in spite of prompt and appropriate management [51]. Its incidence per injection of either bevacizumab, ranibizumab, pegaptanib, or triamcinolone ranges from 0.009% to 1.9% [51–56]. The pathogens most commonly isolated from intravitreal cultures are *Staphylococcus epidermidis* and staphylococci coagulase-negative [57–59]. Optimum management of the ocular surface before, during, and after intravitreal injections remains controversial. A topical combination of povidone-iodine is the only preoperative substance proven in a randomized clinical trial to reduce the risk of endophthalmitis after intraocular surgery [58, 60]. In the 2008 ASRS meeting, it has been suggested that 40% of retina specialists use topical antibiotics before anti-vascular endothelial growth factor intravitreal injections, and 86% use topical antibiotics after anti vascular endothelial growth factor intravitreal injections. In a report from the DRCR.NET, a low rate of endophthalmitis can be achieved by means of a protocol that includes use of topical povidone-iodine, a sterile lid speculum, and topical anesthetic, but does not require topical antibiotics, sterile gloves, or a sterile drape [58]. Achievement of a low rate of endophthalmitis postintravitreal injections does not require topical antibiotic prophylaxis a day before or after the injection [58]. 

## 9. Endophthalmitis after PPV

In a 20-year retrospective review of patients who developed acute onset endophthalmitis after pars plana vitrectomy at Bascom Palmer Eye Institute, 5 out of 6 patients had diabetes. Patients presented with an initial visual acuity ranging from 20/300 to light perception. Visual outcome postsurgery was poor with 4 out of 6 patients having a final vision of light perception or worse. Initial advanced retinopathy stages that these patients had might have hampered any improvement in vision. This is further supported by the Post-Vitrectomy Endophthalmitis Study, where 61% of the patients (11/18) had diabetes mellitus [32]. 

The visual outcome of postvitrectomy endophthalmitis is usually poor [28, 32]. Eyes undergoing sutureless vitrectomy may have an increased risk of infectious endophthalmitis compared to 20-gauge vitrectomy. Series reported earlier suggest that there was an increased risk of endophthalmitis, in contrast to more recent series, which so far report mixed results [31, 34, 61–69]. Meanwhile, variable hypotheses have been advanced to explain why sutureless transconjunctival PPV may lead to a higher rate of postoperative endophthalmitis. Some theories relate it to a lack of complete wound closure. Ultrasound biomicroscopy demonstrates that 25-gauge wounds reappose within 2 weeks [70, 71]. We do not know whether the level of ischemia caused by diabetes interferes in the proper wound healing of the sclera and the development of fibrovascular ingrowth into the vitreous base. Some suggests leaving air- or gas-filled vitreous cavity would allow sclera wound apposition secondary to the surface tension that develops at the wound interface [34, 51, 72]. Others propose that lower infusion rates are a feature of sutureless vitrectomy, and the reduced influx and efflux of fluid may allow a greater bacterial inoculum to remain in the eye [73]. In addition, less vitreous gel is removed during sutureless PPV versus 20-gauge PPV and the residual vitreous skirt may facilitate bacterial adherence and sequester bacteria [74, 75]. Future retrospective or prospective trials need to take into consideration certainly several factors. 

Treatment of postvitrectomy endophthalmitis is analogous to treatment of other types of postoperative endophthalmitis. The best treatment option for gas-filled eyes with postvitrectomy endophthalmitis is not yet established. Intravitreal antibiotics are generally recommended in such eyes, but the dosage and specific drug may vary. The recommended standard dose age of intravitreal antibiotics could be beneficial in even up to 50% of gas-filled eyes [28]. 

## 10. Diabetic Retinopathy and Endophthalmitis

Diabetic retinopathy may become worse with any inflammatory process because eyes in diabetic subjects are susceptible to usually upsurging in some inflammatory factors. Huamonte and associates reported two cases of progression of diabetic retinopathy associated with the inflammatory processes typically associated with sarcoidosis [76]. This concept is further supported by the progression of diabetic retinopathy after cataract extraction. Several authors have shown that retinopathy progression after cataract surgery strongly correlates with the level of preoperative retinopathy [77–82]. Patients with preexisting diabetic retinopathy may be at increased risk for rapid retinopathy progression and a poorer visual outcome after endophthalmitis [37, 83]. So, the visual outcome after endophthalmitis treatment is highly dependent on the level of the damage caused by diabetic retinopathy before the development of endophthalmitis; this prompted the specialists in the field to recommend close monitoring of diabetic patients after endophthalmitis, whether or not they have preexisting DR. 

 In conclusion, although no large-scale study has looked exclusively at subjects with diabetes and endophthalmitis, analysis of the subgroup of patients with diabetes in studies comprising both (patients with diabetes and patients without) suggest that virulence is worse in the former group, growth of organisms is faster and a more aggressive treatment bares a better result. Even though no study has addressed the sutureless trend and the higher risk of endophthalmitis in patients with diabetes, it is wise to consider more conventional wound closures. Special care and more forceful management are warranted in every step. Finally, diabetic retinopathy, if preexisting, progresses to worse, therefore, more frequent retina examinations are advised in this particular subgroup.

## Figures and Tables

**Table 1:** Visual outcome and symptoms in diabetics versus nondiabetics.

	Diabetics	Nondiabetics
Posttreatment visual outcome	(%)	(%)
≥5/200	79.6	90
≥20/100	55.6	77.4
≥20/40	38.9	55.3
Anterior segment characteristics	(%)	(%)
Cataract wound normal	82.8	82.0
Hypopyon	89.7	85.1
Rubeosis	8.6	1.9
Bacterial growth (risk)		
Gram+	Increased	—
Gram−	Same	Same
Virulent organisms	26%	22%
Incidence of retinal detachment (Posttreatment)	6.9%	8.6%
Early procedure performed	20.7%	8.8%
Late procedure performed	31%	27.1%
